# Dynamic monitoring of single-terminal norepinephrine transporter rate in the rodent cardiovascular system: A novel fluorescence imaging method

**DOI:** 10.1016/j.autneu.2019.102611

**Published:** 2020-01

**Authors:** Lily L. Cao, Andrew P. Holmes, Janice M. Marshall, Larissa Fabritz, Keith L. Brain

**Affiliations:** a School of Biomedical Science, Institute of Clinical Sciences, College of Medical and Dental Sciences, University of Birmingham, B15 2TT, United Kingdom; b Institute of Cardiovascular Science, College of Medical and Dental Sciences, University of Birmingham, B15 2TT, United Kingdom; c Department of Cardiology, University Hospitals Birmingham NHS Foundation Trust, Birmingham, United Kingdom

**Keywords:** Norepinephrine transporter kinetics, Fluorescence imaging, Sympathetic, Rodent, Cardiovascular system, NET, norepinephrine transporter, NE, norepinephrine, NTUA, neurotransmitter transporter uptake assay, LAA, left atrial appendage, MA, mesenteric artery

## Abstract

Here, we validate the use of a novel fluorescent norepinephrine transporter (NET) substrate for dynamic measurements of transporter function in rodent cardiovascular tissue; this technique avoids the use of radiotracers and provides single-terminal resolution.

Rodent (Wistar rats and C57BL/6 mice) hearts and mesenteric arteries (MA) were isolated, loaded with NET substrate Neurotransmitter Transporter Uptake Assay (NTUA) ex vivo and imaged with confocal microscopy.

NTUA labelled noradrenergic nerve terminals in all four chambers of the heart and on the surface of MA. In all tissues, a temperature-dependent, stable linear increase in intra-terminal fluorescence upon NTUA exposure was observed; this was abolished by NET inhibitor desipramine (1 μM) and reversed by indirectly-acting sympathomimetic amine tyramine (10 μM). NET reuptake rates were similar across the mouse cardiac chambers. In both species, cardiac NET activity was significantly greater than in MA (by 62 ± 29% (mouse) and 21 ± 16% (rat)). We also show that mouse NET reuptake rate was twice as fast as that in the rat (for example, in the heart, by 94 ± 30%). Finally, NET reuptake rate in the mouse heart was attenuated with muscarinic agonist carbachol (10 μM) thus demonstrating the potential for parasympathetic regulation of norepinephrine clearance.

Our data provide the first demonstration of monitoring intra-terminal NET function in rodent cardiovascular tissue. This straightforward method allows dynamic measurements of transporter rate in response to varying physiological conditions and drug treatments; this offers the potential to study new mechanisms of sympathetic dysfunction associated with cardiovascular disease.

## Highlights

• New method to label noradrenergic terminals in murine cardiovascular tissue ex vivo
• Fluorescent method to monitor NET reuptake rate in rodent heart and arteries
• NET reuptake rate is faster in the heart than arteries, and in mice than rats
• Presynaptic muscarinic receptors may act to reduce NET reuptake rate in the cardiovascular system

## 1 Introduction

Noradrenergic volume transmission depends on the interplay between norepinephrine (NE) release and its subsequent reuptake by the norepinephrine transporter (NET). The presynaptically located NET terminates the action of NE through a Na^+^/Cl^−^-dependent secondary active transport process, which transports NE back into nerve terminals (Iversen, 1963; Esler et al., 1990), and hence represents an important determinant of NE availability in the extracellular space. However, inferences of dynamic changes in NET reuptake rate in mature sympathetic nerve terminals are difficult to achieve, relying on indirect measurements such as NE overflow (with and without NET inhibition) (Esler et al., 1988; Grassi and Esler, 1999) or tissue reuptake of radioactive substrates on a macroscopic scale (Pandit-Taskar and Modak, 2017; Chen et al., 2019).

Tools to directly study NET kinetics in mature sympathetic nerve terminals are needed in order to better understand transporter regulation, which has been shown to be impaired in several cardiovascular diseases (Schroeder and Jordan, 2011, Schroeder and Jordan, 2012), and is frequently used as a target for pharmacotherapeutics (Zhou, 2004). Fluorescence imaging has proved useful in discerning individual synapses microscopically at high temporal resolutions and, indeed, our group demonstrated the use of a fluorescent NET substrate to measure NET function in sympathetic terminals of the mouse vas deferens (Parker et al., 2010). An additional benefit of this technique is the ability to identify such terminals amongst a mix of peripheral neuronal types; for example, in addition to noradrenergic innervation, the heart possesses a combination of cholinergic and nitrergic efferent terminals and sensory nerves (Shivkumar and Ardell, 2016) that are all varicose and morphologically similar (Mitchell, 1953). Being able to simultaneously label for noradrenergic-specific nerves and measure NET transporter function would prove valuable for the understanding of reuptake properties of the NE terminals ex vivo and in vivo. Of particular interest is how NET reuptake rate may change in response to varying pathophysiological conditions, for example in disease states and pharmacological manipulations.

Here, we expand the use of a fluorescent NET substrate to the cardiovascular system, more specifically in the rodent heart and mesenteric arteries, to further understand NET kinetics and its regulation. Using this approach, we studied intra- and interspecies differences in intra-terminal NET transporter kinetics, NET protein temperature dependency as well as beginning an exploration of the effects of autonomic interplay on NE clearance in the heart.

## 2 Material and methods

### 2.1 Ethical approval

All animal care and experimental protocols of this present investigation were undertaken in accord with the ethical guidelines set out by the Animals (Scientific Procedures) Act 1986, National Institutes of Health and the University of Birmingham, United Kingdom. Procedures were performed under UK Home Office license (mouse: PPL PFDAAF77F, rat: PPL PF4C074AD). Animals were housed in individually ventilated cages under standard conditions: 12:12 h light/dark cycle (with light beginning at 0700), 22 °C and 55% humidity. Food and water were available ad libitum.

### 2.2 Animals and tissue isolation

Male and female C57BL/6 mice and male Wistar rats were supplied by Charles River Laboratories (Margate, UK). Animals were rendered unconscious using deep general anaesthesia (3–5% isoflurane in O_2_, flow rate 1.5 L·min^−^^1^). Mouse hearts were subsequently rapidly isolated, while rat hearts and rodent first or second order mesenteric artery/vein bundles were isolated following an approved humane Home Office schedule 1 method: cervical dislocation, confirmation by exsanguination. Tissues were maintained in bicarbonate buffered Krebs-Henseleit solution (KH) containing (mM): NaCl 118, NaHCO_3_ 25, KH_2_PO_4_ 1.2, glucose 11, EDTA 0.5, MgSO_4_ 1.2, CaCl_2_ 2.5, KCl 4.7 and equilibrated with 95% O_2_/5% CO_2_, pH 7.4.

### 2.3 Measurement of norepinephrine transporter rate

To dynamically measure norepinephrine transporter (NET) rate, we utilised the commercially available Neurotransmitter Transporter Uptake Assay (NTUA, R8173; https://www.moleculardevices.com/products/assay-kits/transporters/neurotransmitter-transporter-uptake#gref, San Jose, CA; U.S. Patent # 6420183, 7063952, 7138280 and European Patent # 0906572) kit. NTUA contains a fluorescent substrate for biogenic amine transporters, including NET, and a masking dye that extinguishes extracellular fluorescence.

Rodent cardiac chambers (LAA, left atrial appendage; RAA, right atrial appendage; LV, left ventricle; RV, right ventricle) and mesenteric arteries (MA) were dissected free as described (Holmes et al., 2016; Hansen et al., 2019), transferred to the imaging chamber and securely pinned down as flat as possible onto a Sylgard-lined base. Tissues were continuously superfused at 2 ml·min^−^^1^ with warmed (35–37 °C), oxygenated KH solution for at least 20 min prior to ex vivo studies. The imaging chamber was positioned underneath an upright confocal scanning microscope (Olympus Fluoview FV1000).

The NTUA protocol used throughout was adapted from previous work by our laboratory (Parker et al., 2010). Due to the lack of nerve terminal autofluorescence, tissues were initially superfused with a highly-diluted (1:100) NTUA solution for 10 and 20 min for mouse and rat, respectively, in order to visualise the location of nerve terminals. After this time, three control *z-*stacks of areas of high innervation density consisting of ~15 slices at 1 μm intervals were imaged. In studies to explore the effects of some drugs, there was a 6 min pre-treatment period. Tissues were then exposed to a less-dilute solution of NTUA (1:20) and z-stacks of the same area were imaged every 1 or 2 min. In some experiments, the preparation was returned to KH thereafter in the presence and absence of pharmacological intervention to investigate fluorescence washout in five minute intervals. To demonstrate the overall innervation in this field for visual purposes, *z*-stacks were merged. The imaging software (Olympus FV10-ASW 4.2) was configured to take 512 × 512 pixel images on a one-way mode at 2.0 μs/pixel with wavelength filter set: excitation 405 nm (circa 1% laser power)/emission 460–560 nm with the pinhole as narrow as possible to exclude out-of-focus light.

### 2.4 Image analysis

Image analysis was performed with Fiji (version 1.52a; https://fiji.sc). To quantify the increase in terminal-specific raw fluorescence exerted by NTUA, a region of interest (ROI) was selected around a single nerve terminal at its brightest amongst the first control *z*-stack. The mean fluorescence value was extracted and the same ROI was manually restored in subsequent stacks. To obtain terminal-specific emission intensities, background fluorescence values surrounding the ROI were subtracted from this value.

To measure changes in NET reuptake rate independent of the initial raw fluorescence across experiments (as determined by native uptake of NTUA, confocal detection sensitivity and pinhole diameter), values (1:20, *F*_1:20, *t*_) at each time point, *t*, were normalised to the average of its three control values (1:100, *F*_1:100_) with the equation shown below and the NET reuptake rate was determined by the trendline gradient variable (*y* = *mx* + *c*) of its linear portion:

$$∆F_{1:20,t}=\frac{F_{1:20,t}-F_{1:100}}{F_{1:100}}$$

Similarly, to investigate the decline in fluorescence during washout, the trendline gradient variable was quantified from the fitted linear decline in fluorescence signal.

### 2.5 Fluorescence histochemistry

To visualise intra-terminal catecholamines, rodent hearts and MA were subjected to a sucrose-potassium-glyoxylic acid (SPG) solution (De la Torre, 1980). Hearts were isolated, embedded in cryoprotectant on a cork disc with the cardiac chamber of interest facing up and then quickly frozen in isopentane cooled in liquid nitrogen. Several 10 μm thick cryosections were obtained, transferred to slides and immediately dipped in room temperature SPG solution for 3 s (1 dip/s). Often in the same animal, mesenteric arteries were isolated and immediately submerged in SPG solution for 40 min and stretched out on slides. Tissue slides (heart and vessels) were dried entirely under a cool stream of air facilitated by a desk fan until ground-glass appearance, mounted with mineral oil and heated to 75 °C for 30 min before sealed with a coverslip. The sections were captured with a confocal microscope fitted with an oil objective (Olympus FV1000; 40× 1.3 NA) with wavelength filter set: excitation 405 nm/emission 460–560 nm.

### 2.6 Drugs

NTUA was provided in vials containing its powder form. A total of 10 ml of buffered KH was added to each vial to yield 1:1 NTUA, then split into 50 × 200 μL aliquots and frozen (−20 °C). Desipramine, tyramine, carbachol and atropine (all drugs, Sigma) were made up to 10 mM aliquots with water and also frozen. On the day of use, these aliquots were further diluted with buffered KH to yield the final working concentrations.

### 2.7 Data analysis

Values are expressed as mean ± SEM. Normality of data was checked with a Kolmogorov-Smirnov test. Statistical analysis of normalised data was carried out by unpaired Student's *t-*test, or Welch's correction *t*-test if the variance in data differed; Mann-Whitney test was used as a non-parametric equivalent. Friedman one-way ANOVA test followed by Dunn's multiple comparison test was carried out on data consisting of more than two groups. When reporting percentage differences between datasets, errors of the respective data were combined as the root of the sum of squares of relative errors.

All statistical analyses were carried out on GraphPad Prism (version 8; GraphPad Software Inc., San Diego, CA, USA). *p* < 0.05 was considered statistically significant. Several n abbreviations are used in the text: (i) n is the number of animals and (ii) n_t_ is the number of individual terminals. Statistical analysis was performed on ‘n_t_’ numbers.

## 3 Results

### 3.1 NTUA labelling and histofluorescence staining

**Fig. 1:**
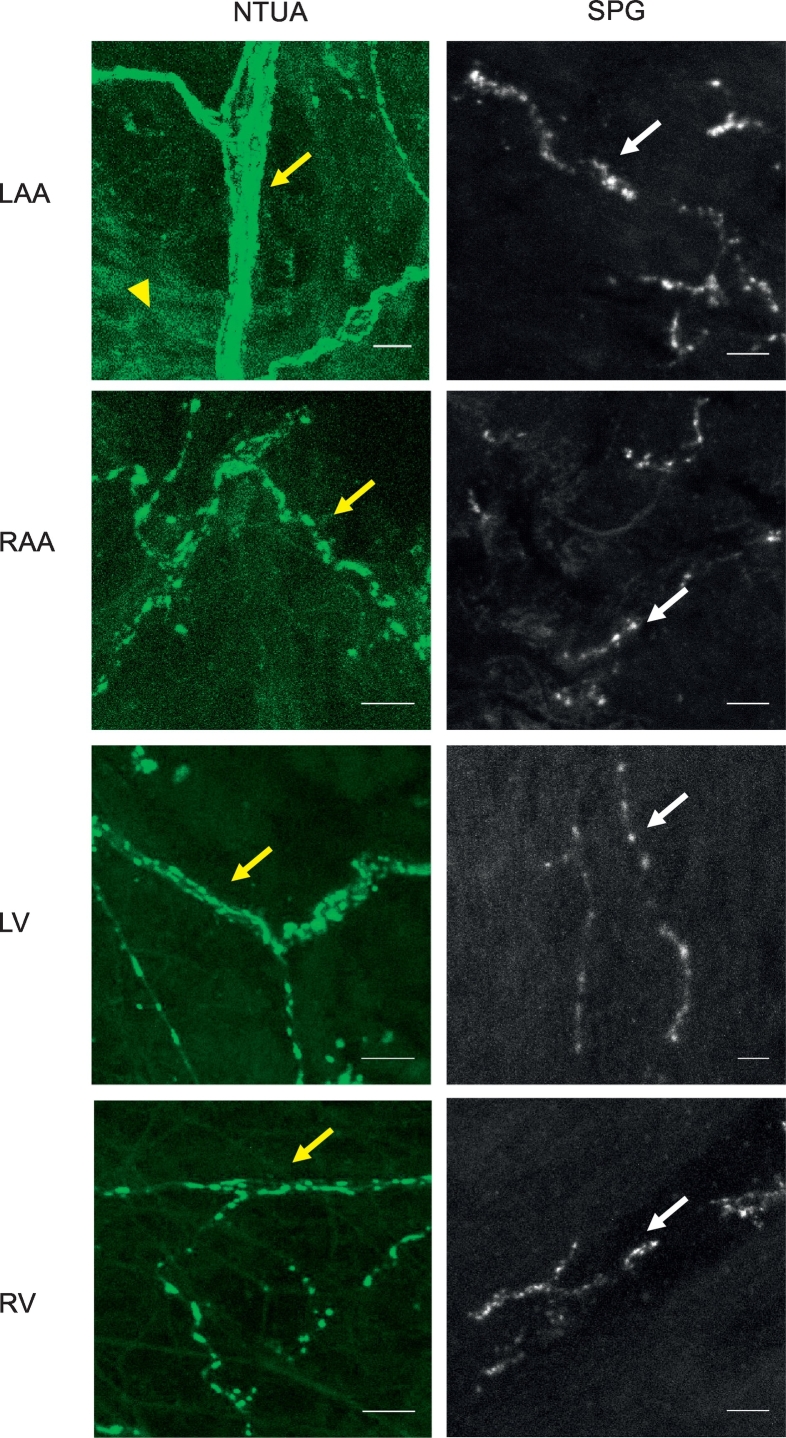
NTUA and SPG labelling of noradrenergic nerves in the rat heart. NTUA labelling (left panels) and glyoxylic acid-induced histofluorescence (SPG; right panels) of each chamber of the rat heart, i.e. left atrial appendage (LAA), right atrial appendage (RAA), left ventricle (LV) and right ventricle (RV). NTUA labelled noradrenergic axonal bundles can be seen dividing off into single fibre axons with punctate swellings of nerve terminals (yellow arrows). The latter was also seen by SPG histofluorescence (white arrows). Yellow arrow heads mark the presence of non-neuronal fluorescence in response to NTUA treatment. For all micrographs, bars = 10 μm.

**Fig. 2:**
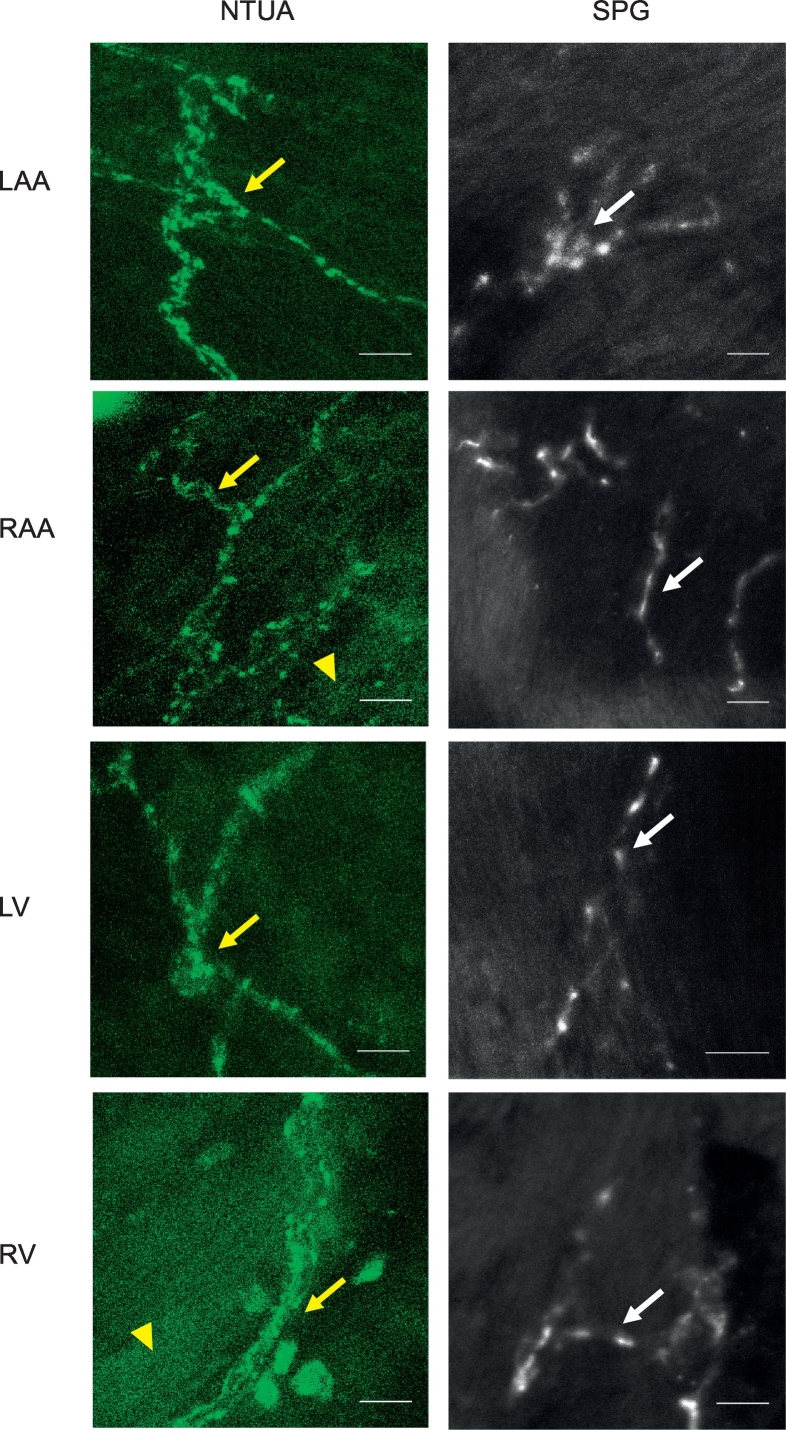
NTUA and SPG labelling of noradrenergic nerves in the mouse heart. NTUA labelling (left panels) and glyoxylic acid-induced histofluorescence (SPG; right panels) of each chamber of the mouse heart, i.e. left atrial appendage (LAA), right atrial appendage (RAA), left ventricle (LV) and right ventricle (RV). NTUA labelling and SPG histofluorescence yielded visualisation of noradrenegic neurones with single fibre axons. Yellow arrow heads mark the presence of non-neuronal fluorescence in response to NTUA treatment. For all micrographs, bars = 10 μm.

**Fig. 3:**
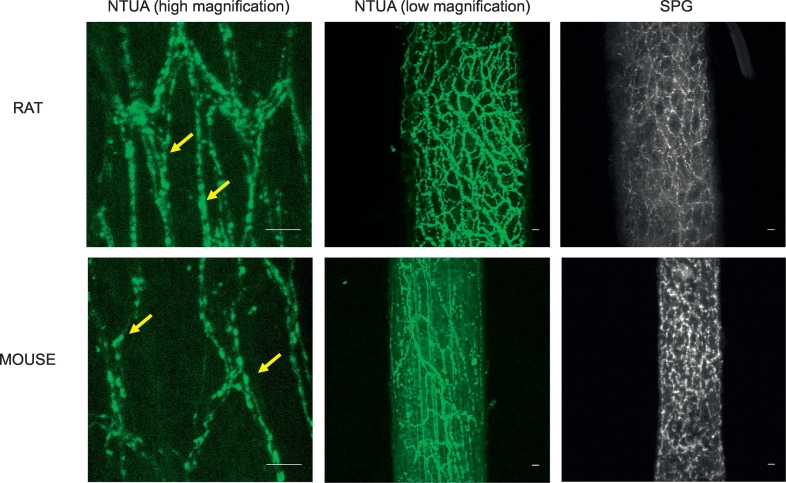
NTUA and SPG labelling of noradrenergic nerves in the rodent mesenteric arteries. At high magnifications, NTUA incubation (left panels; yellow arrows) labelled noradrenergic nerves at the level of single terminals. At low magnifications, NTUA (middle panels) and SPG (right panels) incubation demonstrated perivascular noradrenergic nerves arranged in thick bundles that wraps around the entirety of the vessel. For all micrographs, bars = 10 μm.

Superfusion of rodent cardiac chamber and mesenteric arteries with the NTUA kit revealed networks of sympathetic innervation where coursing axonal bundles can be seen branching out into thinner bundles and ultimately, to thin varicose fibres (left panels – heart: Fig. 1 (rat), Fig. 2 (mouse); left and middle panels – mesenteric artery: Fig. 3). The latter structures are characterised by their classical appearance of ‘beads on a string’ morphology demonstrating the terminal part of the axon. We also loaded NTUA into mouse hearts via Langendorff perfusion (method analogous to Yu et al. (2014), O'Shea et al. (2019)), which resulted in labelling of similar structures (n = 2, *data not shown*). It is interesting to note some non-neuronal fluorescence seen in the NTUA-treated rodent heart (Fig. 1, Fig. 2). Note that under control conditions (prior to adding 1:100 NTUA), no structures resembling nerve terminals were observed.

To demonstrate noradrenergic nerves with an alternative method, we incubated a different set of rodent tissue in a SPG solution. In the heart, this revealed an occasional supply of individual nerve fibres with punctate structures similar to those observed with NTUA. However, axonal bundles were not seen with this method (right panels; heart: Fig. 1 (rat), Fig. 2 (mouse)). Similarly to NTUA labelling, SPG-treated mesenteric arteries resulted in the appearance of very dense plexuses of noradrenergic bundles that innervates the entirety of the vessel. However, while nerve bundles consisting of multiple nerve fibres were evident in NTUA-treated arteries, this was not seen in SPG-treated arteries. In addition to this, nerve structures in the latter treatment appeared ‘scrunched’ compared to their counterparts.

### 3.2 NTUA specificity for NET

**Fig. 4:**
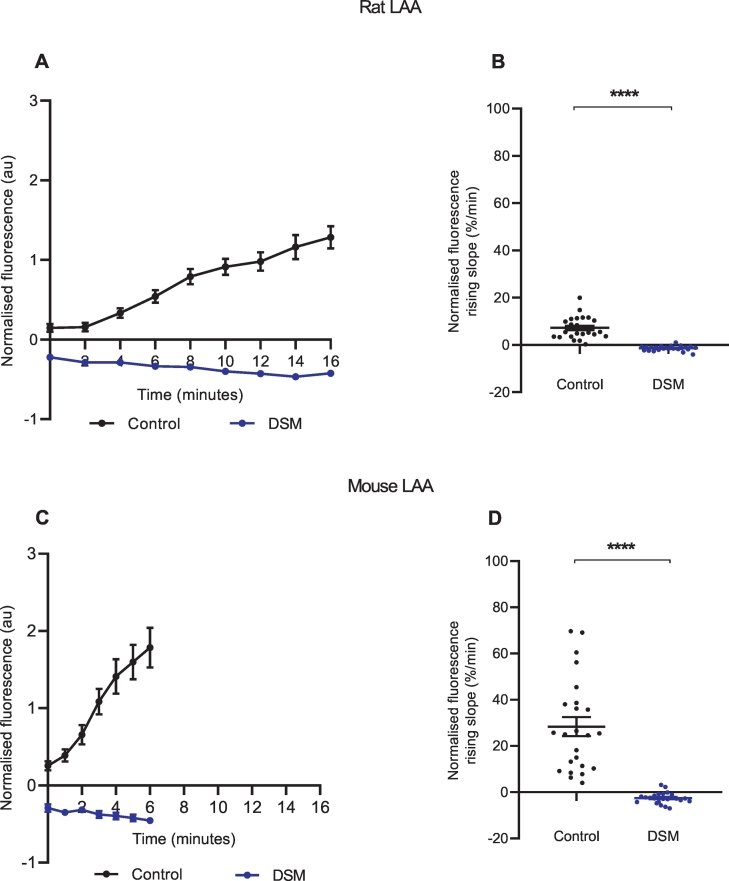
NTUA uptake into noradrenergic nerves is NET-specific in rodent left atrial appendages (LAA). NTUA accumulation into noradrenergic terminals during the 1:20 NTUA test period was completely abolished by tissue pre-treatment with desipramine (DSM, 1 μM). The linear traces in which the NET reuptake rate in the heart was obtained are shown in A (rat) and C (mouse). The gradient variables were extracted and quantified in B (rat) and D (mouse). Rat LAA, control (n = 4, n_t_ = 25) vs. DSM (n = 4, n_t_ = 23). Mouse LAA, control (n = 4, n_t_ = 24) vs. DSM (n = 4, n_t_ = 24). Data presented as mean ± SEM. **** denotes *p* < 0.0001. Welch's *t-*test.

**Fig. 5:**
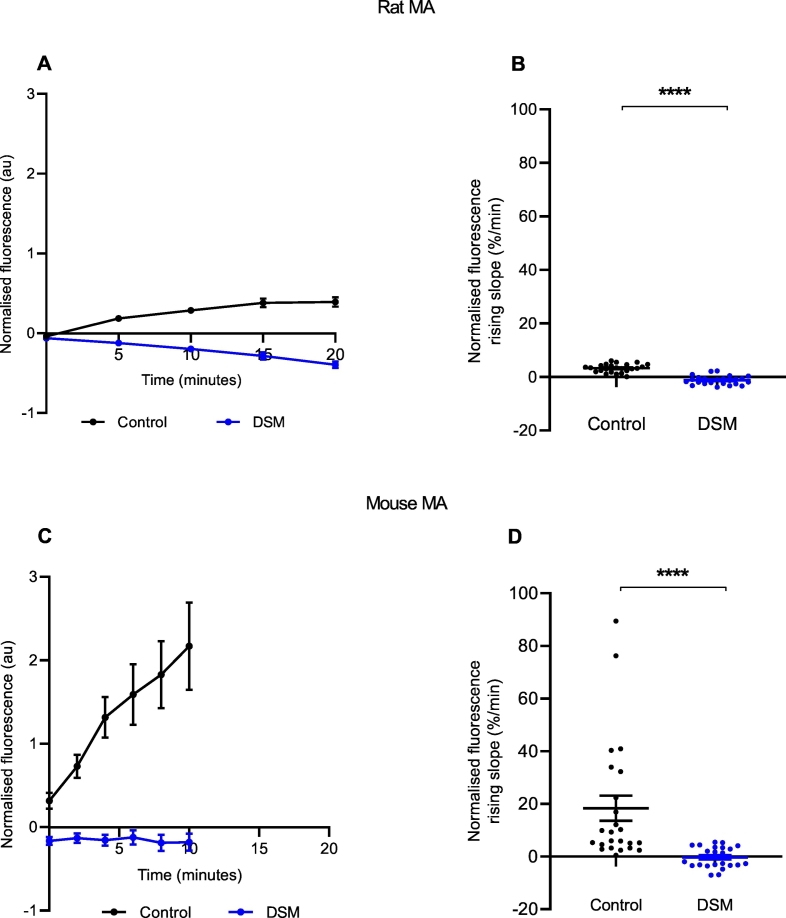
NTUA reuptake into noradrenergic nerves is NET-specific in rodent mesenteric arteries (MA). NTUA accumulation into noradrenergic terminals during the 1:20 NTUA test period was completely abolished by tissue pre-treatment with desipramine (DSM, 1 μM). The linear traces in which the NET reuptake rate in the heart was obtained are shown in A (rat) and C (mouse). The gradient variables were extracted and quantified in B (rat) and D (mouse). Rat MA, control (n = 4, n_t_ = 24) vs. DSM (n_t_ = 24). Unpaired *t-*test. Mouse MA, control (n = 4, n_t_ = 24) vs. DSM (n_t_ = 24). Mann-Whitney *U* test. Data presented as mean ± SEM. **** denotes *p* < 0.0001.

**Fig. 6:**
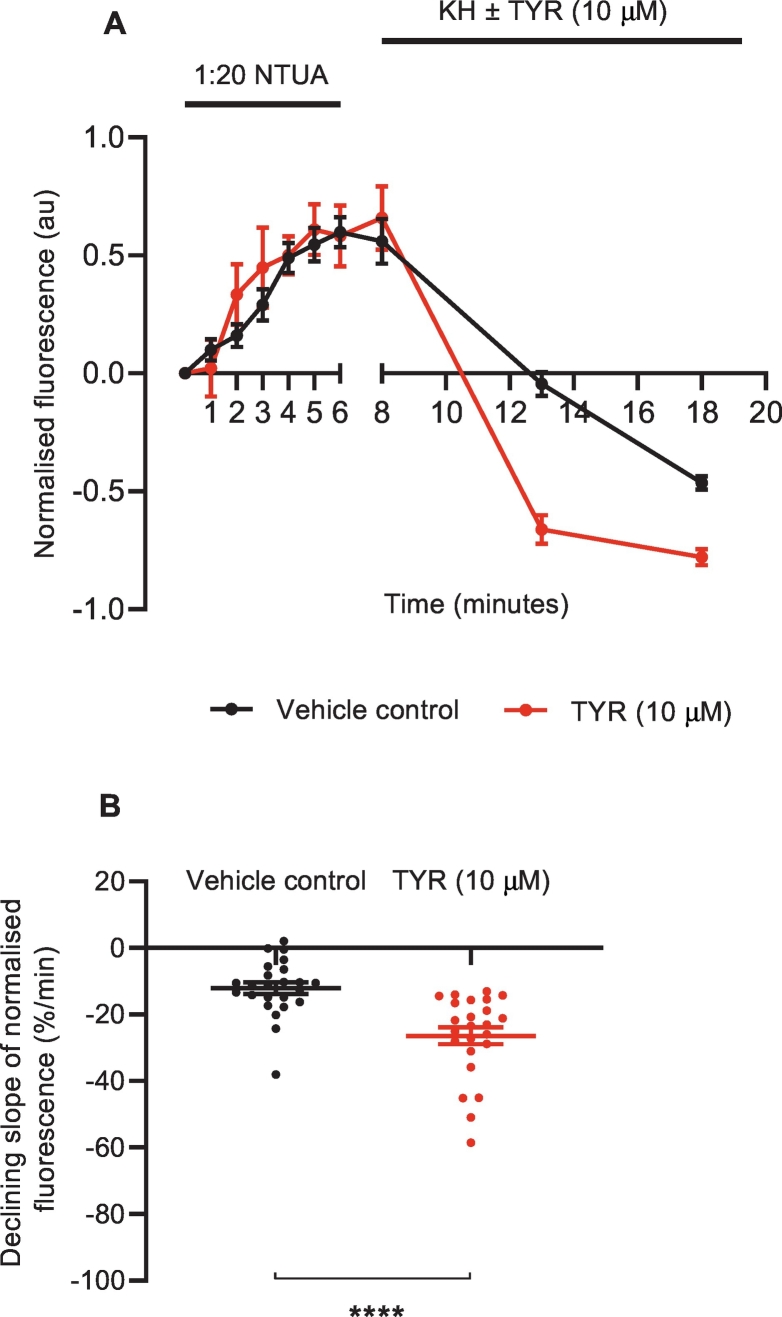
NET substrate tyramine induced rapid loss of intra-terminal fluorescence in mouse LAA. Mouse LAA were subjected to 1:20 NTUA test period for 6 min and promptly returned to KH with or without tyramine (10 μM) and imaged in 5 min intervals. After 5 min, the loss of fluorescence in the TYR group was significantly faster than in the control group. Within 10 min of TYR exposure, fluorescence of some nerve terminals was completely lost as the slope tended towards −1, while they remained visible in the control group (A). The gradient variables were extracted for the first 5 min and quantified in B. Control (n = 4, n_t_ = 24) vs. TYR (n = 4, n_t_ = 24). Data presented as mean ± SEM. **** denotes *p* < 0.0001. Unpaired *t-*test.

To confirm that NTUA uptake into the structures of interest depended on NET, rodent tissues were pre-treated with the NET inhibitor desipramine (1 μM, DSM) for 6 min prior to and during the test period with 1:20 NTUA. This abolished NTUA uptake into nerve terminals during the linear phase in the LAA (rat: control: 7.2 ± 0.9%·min^−^^1^ vs. DSM: −1.4 ± 0.02%·min^−^^1^; *p* < 0.0001 (Fig. 4A, B); mouse: control: 28.4 ± 4.1%·min^−^^1^ vs. DSM: −2.5 ± 0.5%·min^−^^1^; *p* < 0.0001 (Fig. 4C, D)) and MA (rat: control: 3.7 ± 0.3%·min^−^^1^ vs. DSM −1.2 ± 0.3%·min^−^^1^; *p* < 0.0001 (Fig. 5A, B); mouse: control: 18.3 ± 4.8%·min^−^^1^ vs. DSM: −0.3 ± 0.8%·min^−^^1^; *p* < 0.0001 (Fig. 5C, D)). In addition to this, specificity was also investigated with NET reverse substrate tyramine (TYR, 10 μM); following the 1:20 NTUA test period, mouse LAA preparations were returned to KH with or without TYR. In the presence of TYR, the fall in NTUA fluorescence during washout over the first 5 min was 54 ± 5% greater than with KH alone (control: −12.1 ± 1.7%·min^−^^1^ vs. TYR: −26.4 ± 2.5%·min^−^^1^; *p* < 0.0001, Fig. 6B). Thereafter, the tyramine group slope tended towards −1.0 au as individual nerve terminals could no longer be seen while identification remained possible in the control group (Fig. 6A).

### 3.3 Intraspecies and interspecies differences in NET mediated NTUA uptake

**Fig. 7:**
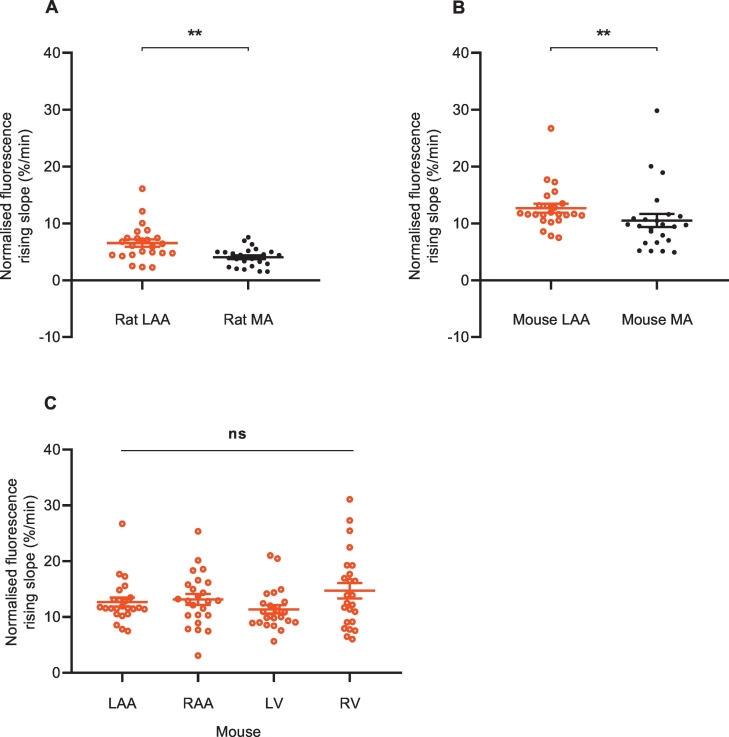
Tissue differences in NTUA uptake rate within each species. NET reuptake rate was faster in the LAA compared to MA in the rat (A) and mouse (B); LAA (n = 4, n_t_ = 24) vs. MA (n = 4, n_t_ = 24). Mann-Whitney *U* test. This did not differ across the mouse heart (C); all four chambers: n = 4, n_t_ = 24, Friedman test followed by Dunn's multiple comparison test. Data presented as mean ± SEM. ** denotes *p* < 0.001, Ns denotes *p* > 0.05.

To investigate intra- and interspecies variation in the NET reuptake rate of singular noradrenergic nerve terminals of rodent cardiovascular tissue, the rates of NTUA uptake in adult animals were compared between LAA and MA within each species and between species. Within each species, the NTUA reuptake rate was consistently faster in the heart than the MA: 62 ± 29% higher in the rat (LAA 6.5 ± 0.6%·min^−^^1^ vs. MA 4.0 ± 0.3%·min^−^^1^; *p* < 0.01, Fig. 7A) and 21 ± 16% higher in the mouse (LAA 12.7 ± 0.8%·min^−^^1^ vs. MA 10.5 ± 1.1%·min^−^^1^; *p* < 0.01, Fig. 7B). We also compared NET reuptake rates across the cardiac chambers in the mouse heart; there were no differences across the atria and ventricles on both the left side and right side (LAA: 12.7 ± 0.8%·min^−^^1^, RAA: 13.2 ± 1.0%·min^−^^1^, LV: 11.4 ± 0.7%·min^−^^1^, RV: 14.7 ± 1.4%·min^−^^1^; *p* > 0.05, Fig. 7C). This suggests a homogenous NET reuptake rate confined to single nerve terminals in the heart, though this is consistently faster than those in the mesenteric arteries.

**Fig. 8:**
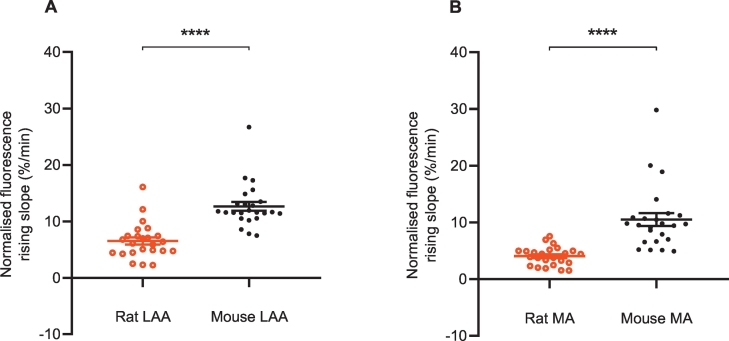
Species differences (rat vs. mouse) in NTUA uptake rate in left atrial appendage (LAA) and mesenteric arteries (MA). In the mouse, NET reuptake rate was faster in both the LAA (A) and MA (B) by approximately two-fold; rat (n = 4, n_t_ = 24) vs. mouse (n = 4, n_t_ = 24). Data presented as mean ± SEM. **** denotes *p* < 0.0001. Mann-Whitney *U* test.

Between species, the NTUA uptake rate was significantly faster by a two-fold difference in the mouse than the rat in both the LAA (Fig. 8A) and the MA (Fig. 8B) by 94 ± 30% and 160 ± 77%, respectively, suggesting for clear species differences in NET reuptake rate.

### 3.4 Temperature-dependent kinetics

**Fig. 9:**
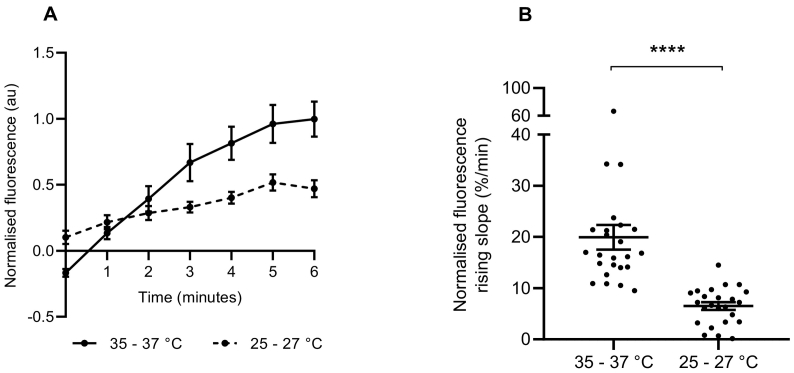
NTUA uptake into noradrenergic nerve terminals is temperature-dependent in the mouse left atrial appendage. The trace of NTUA uptake into terminals was shallower in cooler temperatures during the 1:20 NTUA test period compared to physiological temperatures (A). The gradient variables were extracted and quantified in B. The rate of NTUA uptake was reduced by three-fold when the bath temperature was lowered by 10 °C prior to and during the test period (B) 35–37 °C (n = 4, n_t_ = 24) vs. 25–27 °C (n = 4, n_t_ = 24). Data presented as mean ± SEM. **** denotes *p* < 0.0001. Mann-Whitney *U* test.

To determine if the uptake of NTUA into noradrenergic nerve terminals depended on the rate of carrier-mediated transport rather than passive diffusion, the effect of temperature was investigated in mouse LAA. When the organ bath temperature was cooled by 10 °C for 6 min prior to and during 1:20 NTUA superfusion, the trace of NTUA uptake was shallower (Fig. 9A) the rate of uptake was reduced by nearly three-fold (from control values of 20.0 ± 2.4%·min^−^^1^ to 6.5 ± 0.7%·min^−^^1^; *p* < 0.0001; Fig. 9B).

### 3.5 Muscarinic regulation of NET

**Fig. 10:**
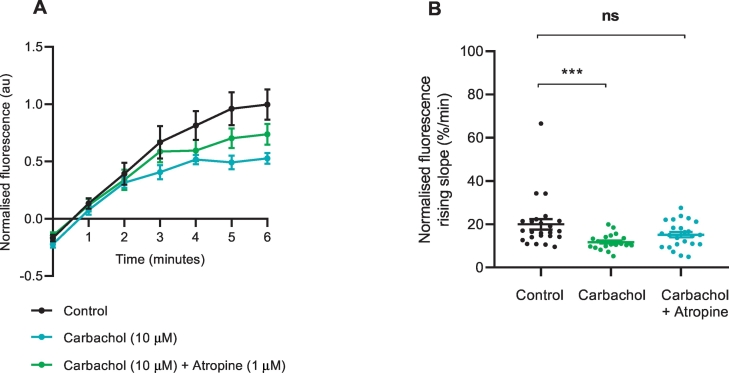
Effect of muscarinic agonist carbachol on NET reuptake rate into noradrenergic terminals of the mouse left atrial appendage. Pre-treatment with muscarinic receptor agonist carbachol (10 μM) reduced NET reuptake rate of NTUA. This was prevented by the inclusion of muscarinic receptor antagonist atropine (1 μM). Control (n = 4, n_t_ = 24) vs. carbachol (n = 4, n_t_ = 24) vs. atropine (n = 4, n_t_ = 24). The gradient variables were extracted and quantified in B. Data presented as mean ± SEM. *** denotes *p* < 0.001, ns denotes *p* > 0.05; Kruskal-Wallis test followed by Dunn's multiple comparison test (B).

To investigate cholinergic influences on NET reuptake rate in the heart, we exposed the mouse LAA to carbachol (10 μM) during the 6 min pre-treatment time and during the 1:20 NTUA test period. This resulted in a reduction in NET-dependent NTUA reuptake rate by 70 ± 36% (control: 20.0 ± 2.4%·min^−^^1^ vs. carbachol: 11.8 ± 0.7%·min^−^^1^; *p* < 0.001; Fig. 10). By pre-treating the tissue with the muscarinic antagonist atropine (1 μM), the NET reuptake rate was recovered to values similar to control (15.1 ± 1.2%·min^−^^1^; *p* > 0.05), indicating functional presynaptic muscarinic receptors on noradrenergic nerve terminals.

### 3.6 Susceptibility to photobleaching

**Fig. 11:**
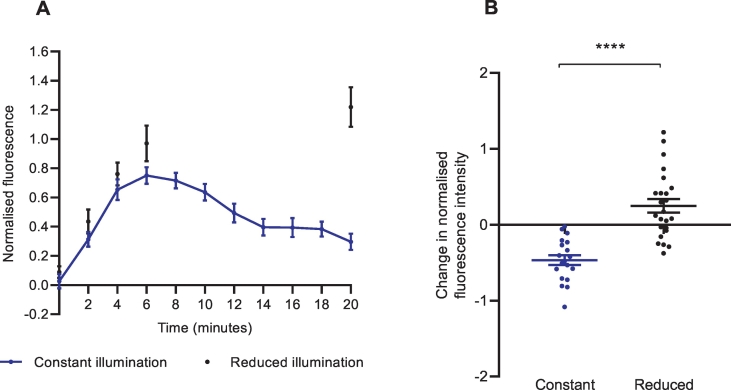
Frequent imaging protocols resulted in an attenuation of NTUA-induced fluorescence intensity in nerve terminals of the mouse left atrial appendage. (A) Timeline of NTUA-induced fluorescence in different imaging conditions. Constant imaging conditions (2-minute intervals for 20 min) resulted in an attenuation of normalised fluorescence intensity between t = 6 and t = 20 (n = 4, n_t_ = 19); the opposite was true when an absence of imaging occurred at *t* = 8–18 (n = 4, n_t_ = 24). (B) The change of normalised fluorescence intensity between t = 6 and t = 20 in different imaging conditions. The constant imaging conditions tended towards a negative change in normalised fluorescence intensity whereas there a positive change for the reduced imaging conditions. Constant (n = 4, n_t_ = 19) vs. reduced (n = 4, n_t_ = 24), unpaired Student's *t-*test. Data presented as mean ± SEM. **** denotes *p* < 0.0001.

As the investigation of intrinsic NET reuptake rate depended on frequent imaging protocols, we were interested in the photostability of the NTUA fluorescence in the mouse LAA. Under constant imaging conditions every 2 min for 20 min, NTUA fluorescence steadily attenuated over time. However, in reduced imaging conditions consisting of absent illumination for 10 min, fluorescence was maintained at significantly higher values at the protocol endpoint (Fig. 11A); we quantified this as the change in fluorescence between *t* = 6 and *t* = 20 (constant: −0.47 ± 0.07 vs. reduced: 0.25 ± 0.09, *p* < 0.0001; Fig. 11B).

## 4 Discussion

### 4.1 The use of fluorescence to monitor transporter activity

Literature reports of norepinephrine transporter (NET) kinetics for the past three decades have relied heavily on the use of radiotracers in cells (Raffel et al., 2013), animals (Lopez-Picon et al., 2019; Chen et al., 2019) and humans (Wichter et al., 2002; Pandit-Taskar and Modak, 2017). Unfortunately, in addition to the disadvantages associated with nuclear safety, expenses and requirement for specialist personnel, the data obtained on real-time changes in NET regulation and function are limited and confined to the macroscopic scale. However, during this time, fluorescence imaging diversified and the use of common dyes in the field of NETs was developed, including stilbazolium dyes, for example 4-[4-(dimethyl amino)styryl]-*N*-methylpyridinium iodide (ASP^+^; analogue of neurotoxin, MPP^+^) (Haunso and Buchanan, 2007; Wilson et al., 2012) and false fluorescent neurotransmitters (Dunn et al., 2018), which both provide the ability to discern individual terminals microscopically in living tissue.

In this present study, we adapted the use of a novel and commercially available fluorescent kit, Neurotransmitter Transporter Uptake Assay (NTUA), to monitor NET reuptake rate in rodent heart and blood vessels at the spatiotemporal resolution of single noradrenergic nerve terminals. So far, this technique has been validated in cell culture systems (Jorgensen et al., 2008), including cardiac stellate sympathetic neurones (Shanks et al., 2013), as well as the intact mouse vas deferens (Parker et al., 2010). We demonstrate, for the first time, the use of this method in rodent cardiovascular tissue ex vivo and its applicability to investigate changes in NET function in response to functional manipulations, such as temperature- and drug-induced effects.

### 4.2 NTUA as a labelling method for sympathetic nerve terminals in cardiovascular tissue

The nerve terminal branches seen upon exposure to NTUA superfusion are structurally similar to sympathetic nerve terminals described elsewhere (Rysevaite et al., 2011; Yokomizo et al., 2015) and as seen with glyoxylic acid labelling (as we show here). These punctate structures are joined by intervaricose segments and are indicative of synapse formation (Bennett, 1993; Brain et al., 1997); tracing them backwards, they combine with others to form small bundles of fibres, which ultimately form large nerve bundles. In the heart, the latter structures and single nerve fibres have been found in the epicardium and close to cardiomyocytes (Kawano et al., 2003), respectively. However, this exact distribution was difficult to determine as it was challenging to flatly secure the tissue in the superfusion bath. Additionally, NTUA labelling could only be seen to a depth of a few tens of micrometers, which is likely to have been limited by the depth penetration of the microscope under the current recording conditions (Pawley, 2006). We also cannot rule out a failure of the dye to penetrate deep into the tissue from the epicardial surface. To overcome this, we have also achieved labelling of nerve terminals using Langendorff perfusion; while this is helpful for studies that require the mere identification of sympathetic innervation, it is less suitable for the study of kinetics due to difficulty of imaging while perfusing. Interestingly, in the heart, NTUA superfusion led to increases in fluorescence of non-neuronal structures, similar to that described in the mouse vas deferens (Parker et al., 2010). However, this was not problematic for nerve terminal identification as its morphology was distinct and fluorescence was significantly brighter than non-neuronal fluorescence.

Significant advantages of NTUA labelling over histochemical staining are that it does not require tissue cryosection, which could have prevented the visualisation of axonal bundles in this study, and it can be used as an intravital stain. NTUA labelling does not suffer from the disadvantage of shrinkage artefacts induced by dehydration that occurs with fixation methods, including glyoxylic acid labelling. Such shrinkage may explain why the glyoxylic acid-treated terminals of rodent mesenteric arteries appear compressed. This also led to difficulties in identifying individual nerve fibres that constitute nerve bundles, unlike those labelled with NTUA (compare image panels in Fig. 3). Shrinkage artefacts prevent accurate nerve density quantification even with stretched preparations (Furness and Costa, 1975) and it is difficult to count individual fibres in bundles. Therefore, in addition to the advantages of NTUA for ex vivo studies, it also maintains physiological nerve architecture and structure.

### 4.3 Mechanisms of NTUA uptake

Transport by NET (and other monoamine transporters) is driven by a Na^+^/Cl^−^-secondary active process (Apparsundaram, 2001) and uptake has been shown to be temperature-dependent with ASP^+^ (Schwartz et al., 2003; Mason et al., 2005) and ^3^H-NE (Inazu et al., 2003). Here, we show a three-fold reduction in NTUA uptake rate in cooler temperatures (25–27 °C) compared to physiological temperatures. This is similar to the Q_10_ (ratio between transport activity at two temperatures differing by 10 °C) value of 2.5 previously reported for ASP^+^ transport by hNET (Mason et al., 2005), thus strongly suggesting carrier-mediated (rather than passive) uptake in this study.

The NTUA assay is also a substrate for the dopamine- and serotonin transporter (DAT and SERT, respectively). Whilst DAT expression and function has been detected in the guinea pig heart (Palomar et al., 2011) and in the rabbit celiac artery (Amenta and Ferrante, 1983), respectively, there is currently no evidence of its existence in rodent cardiovascular tissue (Mitsuma et al., 1998). In addition to this, desipramine is a more potent inhibitor of NET (K_i_ = 7.36 nM) than DAT (K_i_ > 10,000 nM) (Zhou, 2004), thus the complete abolition of NTUA uptake in this study suggests against the role of DAT in NTUA uptake. SERT, on the other hand, has been consistently observed in rodent cardiovascular structures, for example valves (Gustafsson et al., 2005; Pavone et al., 2008) and endocardium (Mekontso-Dessap et al., 2006), and arterial smooth muscle (Ni et al., 2004). However, SERT in these studies were shown to be present in valve interstitial cells, the innermost layer of cardiac chamber lining and nerve independent, respectively. As we focused on the uptake of NTUA into neuronal structures present in the subepicardium and on the surface of perivascular nerves, the contribution of SERT to NTUA uptake was also unlikely. Therefore, NET is most likely uptake mechanism of NTUA in rodent heart and mesenteric artery.

To further support for NET specificity, we next hypothesised faster loss of intra-terminal NTUA fluorescence during washout in the presence of a NET reverse substrate. Here, tyramine, a NET substrate that stimulates peripheral release by reverse transport through NET (Pei et al., 2016), led to an over two-fold uniform decline in NTUA fluorescence during the washout phase compared to the control group. Tyramine releases monoamines from vesicular stores (Trendelenburg et al., 1987); it appears that the rapid loss of NTUA fluorescence in this study was mediated by a release of NTUA into the cytoplasm followed by extrusion by the reversal of the NET transporter. This was also previously demonstrated in the mouse vas deferens where storage was reversed by amphetamine, another indirectly-acting sympathomimetic amine (Parker et al., 2010). The use of a VMAT and NET transporter substrate to deplete NTUA fluorescence further supports the idea that NTUA is stored in sympathetic terminals and their vesicles.

### 4.4 Intraspecies differences in NET reuptake rate

Having demonstrated NTUA uptake is NET-specific, we determined if NET reuptake rate differed between organs and within different parts of the heart. We show that, in both the rat and mouse, nerve terminals present in the heart exhibit faster NET reuptake rate than those in the mesenteric arteries, suggesting that norepinephrine (NE) reuptake rate differs between these tissues. A similar difference was reported previously in the somas of isolated rat postganglionic neurones from the cardiac stellate ganglia compared to those from the superior mesenteric ganglia (Shanks et al., 2013). Additionally, on a macroscopic scale, the heart has been shown to exhibit a greater dependence on NET for NE removal compared to other organs in mice (Krell and Patil, 1972), rats (Thackeray et al., 2007), monkeys (Farde et al., 1994) and humans (Goldstein et al., 1988). This functional asymmetry is important as it implies differences in cellular signalling and in autonomic regulation between tissues. Indeed, one study found selective impairment of NET in cardiac stellate postganglionic neurones in SHRs compared to WKYs (Shanks et al., 2013). However, whether this is due to a higher NET expression density, NET variants or faster transport kinetics of individual NET in these tissues is currently unknown and to our knowledge, there are no studies that directly compare this.

In the mouse heart, there appeared to be similar terminal-specific NET reuptake rates across each cardiac chamber. A previous study also demonstrated homogeneous reuptake of radiolabelled NE in the grouped mouse atria and ventricles (Shirey-Rice et al., 2013), thus suggesting for cardiac symmetry in NET reuptake rate. However, there are also separate reports of highest NET reuptake rate found in the left ventricle (Wehrwein et al., 2008) and right ventricle (Raffel et al., 2006). Those studies, however, quantify total tissue NET reuptake rate rather than terminal-specific reuptake, as we have shown here; the latter may collectively sum up to macroscopic differences, though this requires further study. Most importantly, tissue reuptake also depends on the density of terminals and tissue volume. To our knowledge, there are currently no other studies that have studied intraspecies differences in terminal-specific NET reuptake rate across the heart.

Heterogeneous NET reuptake rates in the left ventricle have been reported in various species (Chilian et al., 1982; Munch et al., 2005; Kusmic et al., 2019), including rodents (Kiyono et al., 2002; Ieda et al., 2007), although in humans it appears as homogeneous except in disease states (Beau and Saffitz, 1994; Kiyono et al., 2001; Wichter et al., 2002). This is thought to contribute to electrical heterogeneity and thus arrhythmogenesis found in heart failure (Beau and Saffitz, 1994) and Brugada syndrome patients (Wichter et al., 2002). In this study, we made no attempt to quantify NET reuptake rate throughout the left ventricle. However, we present a tool for others to investigate cardiac heterogeneity further for distinct populations of sympathetic nerve terminals throughout the heart. This would prove clinically relevant as sympathetic defects are linked to sudden death in these patients and offers an opportunity to address important mechanistic questions.

### 4.5 Interspecies differences in NET reuptake rate

In this study, we also show a two-fold faster NET reuptake rate in the mouse than rat in both heart and vascular tissue. To our knowledge, research into interspecies differences in transporter function have so far been studied very rarely. Currently, only one group has investigated such differences and, similar to our study, also found a two-fold faster reuptake rate in perfused isolated whole hearts in the mouse than rat (Iversen et al., 1971). While another group have also investigated NE reuptake in hearts of rats (Krell and Patil, 1972) and mice (Garg et al., 1973), these took place as separate studies and differed in their technical and analytical methods, thus their respective results cannot be directly compared. One explanation for interspecies differences is the dissimilarity of NET transporter kinetics. While rat NET have been shown to have 90% amino acid identity to its mouse counterparts (Ensembl database (Zerbino et al., 2017)), there are no studies that directly compare their functional kinetic differences; however, this has been shown to be present between rat-, bovine- and human-NET (Paczkowski et al., 1999). To better understand the results of this present study, future work could investigate individual transporter function (substrate molecules per protein per second) across species, as described elsewhere (Schwartz et al., 2003). Nevertheless, this current study is the first to compare interspecies differences in terminal-specific NET reuptake rate between the two most commonly used species in biomedical research.

### 4.6 Muscarinic inhibition of NET rate in the heart

Sympathetic postganglionic neurones are endowed with heteroreceptors that modulate neurotransmitter release (Schlicker and Göthert, 1998). In the present study, we explored the effects of one possible heteroreceptor on NE clearance mechanisms. We found a muscarinic (carbachol)-mediated reduction in NET reuptake rate, similar to that described previously in the mouse vas deferens (Parker et al., 2010). Studies with isolated guinea pig chromaffin cells have also shown reduced NE reuptake with acetylcholine (Role and Perlman, 1983). In SK-N-SH cells, this effect has been shown to be governed by a protein kinase C (PKC)-dependent mechanism (likely M_3_ receptor subtype) (Apparsundaram et al., 1998a), which also parallels findings with PKC activator β-PMA (Apparsundaram et al., 1998b). The muscarinic-NET effect may be facilitated by NET/syntaxin 1A interactions as the PKC-mediated disruption caused by muscarinic agonists in CHO-M_3_ cells led to an increased propensity for NET redistribution and a parallel decrease in intrinsic activity (Sung et al., 2003). Whether or not this effect in the present study was mediated by M_1_ or M_3_ receptors (two subtypes coupled to G_q_ in whole heart tissue (Saternos et al., 2018)) is currently unknown.

Muscarinic inhibition of norepinephrine release has been well-investigated in the cardiovascular system (Fozard and Muscholl, 1972; Manabe et al., 1991) and it has been interpreted as a source of cross-inhibition from parasympathetic to sympathetic terminals (Ritter et al., 2020). Here, we demonstrate a muscarinic inhibition of NET reuptake rate, the functional effect of which would be to amplify noradrenergic neurotransmission. A simple interpretation of this is that the inhibition of NET reuptake partially negates or even reverses the cross-inhibition of release. The relative importance of such a mechanism in the presence of exocytosis and reuptake inhibition may depend on factors such as the spatiotemporal release of both acetylcholine and norepinephrine at once in the heart. For example, during periods of strong sympathetic and parasympathetic co-activation (“autonomic conflict”) such as cold-water submersion (Paton et al., 2005), there is evidence to demonstrate its tendency for arrhythmogenesis (Winter et al., 2018), thus we speculate that such co-activation coupled with attenuated NET function might lead to an overall pathological augmentation of noradrenergic-driven electrical instability to the heart.

Despite these explanations however, we cannot rule out the possibility of an alternative mechanism of action: carbachol acting post-junctionally to release mediators for retrograde signalling to the nerve terminals. Such retrograde signalling following muscarinic receptor activation has been shown to be important in the brain (Thomsen et al., 2018), but its role in the periphery, including the heart, has not yet been explored. Therefore, although the direct action of carbachol on the nerve terminals here seems speculative, at present it is the most parsimonious explanation for the current results.

### 4.7 Photosensitivity of NTUA

To demonstrate the photosensitivity of NTUA, we investigated the effect of constant illumination vs. reduced illumination protocols. We found that with reduced illumination, the attenuation of fluorescence signal following the initial linear rise in the minute-by-minute protocol was diminished, thus highlighting the experimental limitation associated with fluorescence imaging. This questions the present study design and whether monitoring NET reuptake of NTUA at each minute, rather than just the start- and end-point, was necessary. However, as this study focused on the use of a fluorescent substrate to dynamically monitor transporter kinetics, frequent illumination protocols were essential. Despite this, there is scope for future studies to identify key time points and image as necessary to prolong the overall duration of imaging and minimise perturbations to physiological processes. Researchers should also take care of accurate protocol reporting and adherence to imaging parameters for experimental replication.

## 5 Future directions

We have optimised a new optical technique to dynamically monitor NET reuptake rate in the heart and blood vessel ex vivo. This should prove valuable for studies of sympathetic nerve function under various phenotypes, e.g. hypertensive rats, and/or various conditions, for example different pharmacological agents; ultimately, allowing the mechanisms underlying these conditions to be elucidated. Previous work with SHRs found impairment of NET reuptake rate in the soma of isolated cardiac postganglionic neurones (Shanks et al., 2013) compared to WKY controls; with this present technique it would be interesting to see if this is consistent at neuroeffector sites at the level of the tissue. Moreover, extending this technique to an in vivo setting would be of interest to allow for investigations of central and local paracrine effects on NET transporter regulation.

## 6 Conclusion

To conclude, the overall significance of this present study is the validation of a new fluorescent tool to measure intra-terminal NET function at the site of action in cardiovascular tissue ex vivo. With this method, we found symmetrical NET reuptake rates throughout the mouse heart, which were significantly faster than vascular tissue; NET reuptake kinetics were also consistently faster in mouse than rat, i.e. the two most used species in biomedical research. We also began to explore muscarinic drug action on NET to investigate possible influences of parasympathetic regulation on NE clearance. We hope this new technique will help unveil important mechanisms that regulate NET in varying physiological conditions, drug treatments and disease states. Finally, we predict that it will be a valuable tool in order to reveal new mechanisms of sympathetic dysfunction associated with cardiovascular disease.
